# Bis(butane-1,4-diammonium) di-μ-oxido-bis[trifluoridooxidomolybdate(V)] monohydrate

**DOI:** 10.1107/S1600536812046892

**Published:** 2012-11-24

**Authors:** J. Lhoste, A. Hemon-Ribaud, V. Maisonneuve, S. Jobic, M. Bujoli-Doeuff

**Affiliations:** a LUNAM Université, Université du Maine, UMR 6283 CNRS, Institut des Molécules et des Matériaux du Mans, Avenue Olivier Messiaen, 72085 Le Mans Cedex 9, France; b Institut des Matériaux Jean Rouxel, UMR 6502 CNRS, 2 rue de la Houssinière, 44322 Nantes Cedex 3, France

## Abstract

The title compound, (C_4_H_14_N_2_)_2_[Mo_2_O_4_F_6_]·H_2_O, was obtained by solvothermal reaction at 443 K for 72 h from a mixture of MoO_3_, HF, 1,4-diamino­butane (dab), water and ethyl­ene glycol. The structure consists of [Mo_2_O_4_F_6_]^4−^ anionic dimers containing strongly distorted MoO_3_F_3_ octa­hedra (with twofold symmetry), diprotonated dab cations and water mol­ecules (twofold symmetry) in the ratio 1:2:1. The cohesion of the three-dimensional structure is ensured through N—H⋯O, N—H⋯F and O—H⋯F inter­actions.

## Related literature

For background to the physical-chemical properties of hybrid compounds, see: Nakajima *et al.* (2000 ▶). For related structures containing discrete entities, see: Mattes & Lux (1976 ▶); Mattes *et al.* (1980 ▶); Chakravorti & Bera (1983 ▶); Adil *et al.* (2007 ▶); Aldous & Lightfoot (2012 ▶).
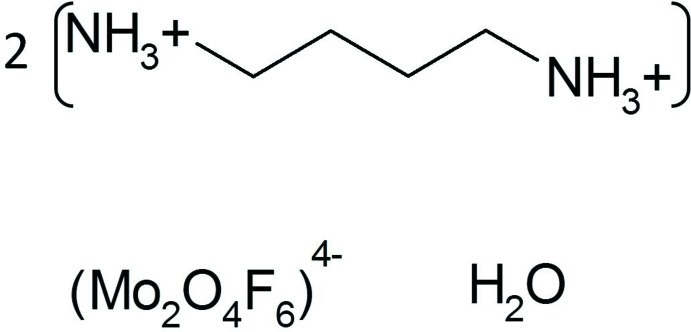


## Experimental

### 

#### Crystal data

(C_4_H_14_N_2_)_2_[Mo_2_O_4_F_6_]·H_2_O
*M* *_r_* = 568.24
Monoclinic, 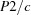

*a* = 8.010 (2) Å
*b* = 8.788 (2) Å
*c* = 14.294 (4) Å
β = 103.019 (12)°
*V* = 980.3 (4) Å^3^
*Z* = 2
Mo *K*α radiation
μ = 1.36 mm^−1^
*T* = 296 K
0.15 × 0.13 × 0.03 mm

#### Data collection

Bruker APEXII diffractometer
Absorption correction: multi-scan (*SADABS*; Bruker, 2001 ▶) *T*_min_ = 0.816, *T*_max_ = 0.960
35088 measured reflections
3240 independent reflections
2690 reflections with *I* > 2σ(*I*)
*R*_int_ = 0.036

#### Refinement

*R*[*F* ^2^ > 2σ(*F* ^2^)] = 0.030
*wR*(*F* ^2^) = 0.078
*S* = 1.05
3240 reflections
135 parameters
1 restraint
H atoms treated by a mixture of independent and constrained refinement
Δρ_max_ = 1.40 e Å^−3^
Δρ_min_ = −0.65 e Å^−3^

### 

Data collection: *APEX2* (Bruker, 2007 ▶); cell refinement: *SAINT-Plus* (Bruker, 2007 ▶); data reduction: *SAINT-Plus*; program(s) used to solve structure: *SHELXS97* (Sheldrick, 2008 ▶); program(s) used to refine structure: *SHELXL97* (Sheldrick, 2008 ▶); molecular graphics: *ORTEP-3* (Farrugia, 2012 ▶) and *DIAMOND* (Brandenburg, 1999 ▶); software used to prepare material for publication: *enCIFer* (Allen *et al.*, 2004 ▶).

## Supplementary Material

Crystal structure: contains datablock(s) I, global. DOI: 10.1107/S1600536812046892/vn2059sup1.cif

Structure factors: contains datablock(s) I. DOI: 10.1107/S1600536812046892/vn2059Isup2.hkl

Additional supplementary materials:  crystallographic information; 3D view; checkCIF report

## Figures and Tables

**Table 1:** Selected bond lengths (Å)

Mo1—O1	1.9754 (18)
Mo1—O1^i^	1.9642 (18)
Mo1—O2	1.7058 (19)
Mo1—F1	2.0786 (17)
Mo1—F2	2.0612 (17)
Mo1—F3	2.1666 (15)

Symmetry code: (i) 
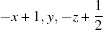
.

**Table 2:** Hydrogen-bond geometry (Å, °)

*D*—H⋯*A*	*D*—H	H⋯*A*	*D*⋯*A*	*D*—H⋯*A*
N1—H1*D*⋯O1*W* ^ii^	0.96 (4)	1.93 (4)	2.889 (3)	172 (3)
N1—H1*E*⋯F3	0.87 (4)	1.94 (4)	2.760 (3)	158 (4)
N1—H1*F*⋯F2^iii^	0.88 (4)	1.80 (4)	2.679 (3)	178 (4)
N2—H2*D*⋯F1^iv^	0.93 (4)	1.87 (4)	2.779 (3)	167 (3)
N2—H2*E*⋯F3^v^	0.91 (4)	1.94 (4)	2.820 (3)	161 (3)
N2—H2*F*⋯O1^vi^	0.91 (4)	2.00 (4)	2.865 (3)	159 (3)
O1*W*—H1*W*⋯F1	0.90 (1)	1.82 (1)	2.711 (3)	178 (4)

Symmetry codes: (ii) 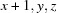; (iii) 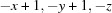; (iv) 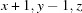; (v) 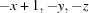; (vi) 
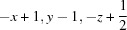
.

**Fig. 1.**
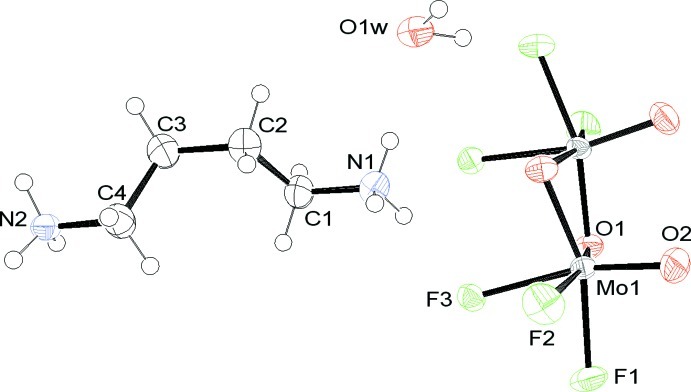
ORTEP view (Farrugia, 2012) of the water molecule, [H2dab]2+ cation and (Mo2O4F6)4- anion in [H2dab]2.(Mo2O4F6).H2O. Atomic displacement ellipsoids are shown at the 50% probability level.

**Fig. 2.**
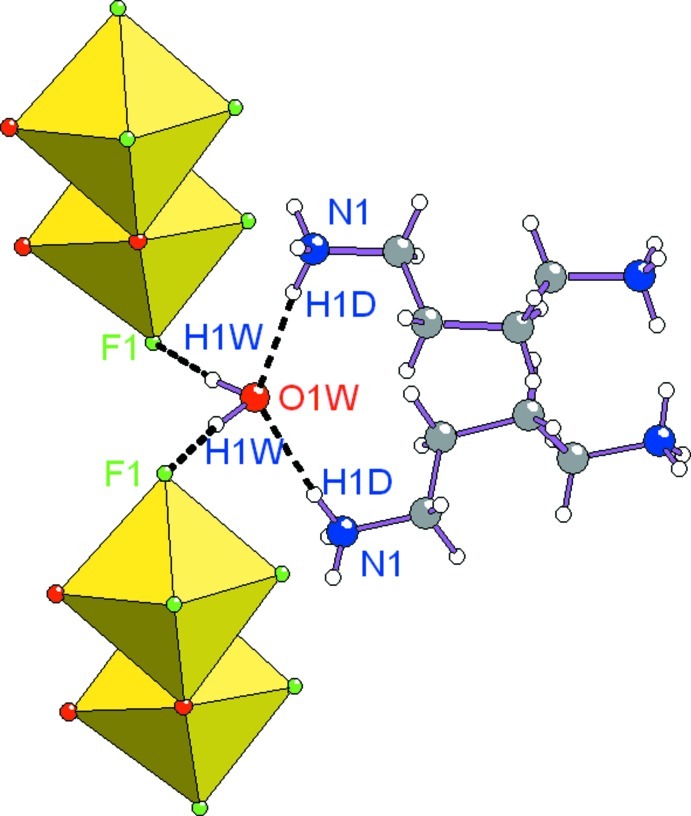
Environment of the water molecule in [H2dab]2.(Mo2O4F6).H2O. O—H···F and N—H···O bonds are shown as dashed lines.

**Fig. 3.**
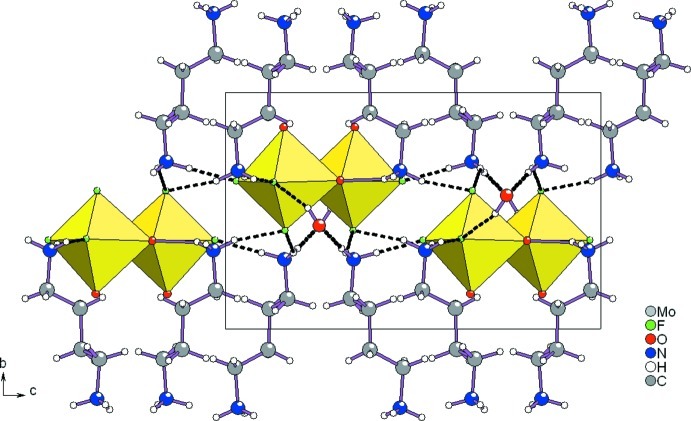
[100] view of the crystal packing of [H2dab]2.(Mo2O4F6).H2O.

## supplementary crystallographic information

### Comment

Transition-metal oxofluoride hybrids have been intensively studied due to their interesting magnetic, optical and electrochemical properties (Nakajima *et al.*, 2000). This paper presents a new organic-inorganic hybrid compound with molybdenum (V) and 1,4-butanediamine. To date, few hybrid oxofluoromolybdates (V) are reported in the literature. [MoOF_5_]^2-^ monomers, [Mo_2_O_2_F_9_]^3-^ and [Mo_2_O_4_F_6_]^4-^ dimers were obtained with ammonium by Mattes *et al.* (1976, 1980). Adil *et al.* (2007) reported the previous monomer with tren cations. Two other compounds, built up from [Mo_2_O_4_F_4_]^2-^ dimers with bipyridinium or phenanthrolinium, were synthesized by Chakravorti *et al.* (1983). It must be noted that the same author obtained many alkali metal molybdenum (V) complexes; their structures involve [MoOF_5_]^2-^, [Mo_2_O_4_F_4_]^2-^, [Mo_2_O_4_F_5_]^3-^ and [Mo_2_O_4_F_6_]^4-^ anions. Recently, a novel tetrameric unit [Mo_4_O_8_F_10_]^6-^ was observed in (NH_4_)_6_[Mo_4_O_8_F_10_] and K_6_[Mo_4_O_8_F_10_] (Aldous & Lightfoot, 2012).

The structure of a new oxofluoride molybdate [H_2_dab]_2_.(Mo_2_O_4_F_6_).H_2_O, synthesized in the MoO_3_-dab-HF_aq_-water-ethylenglycol system, is here described. It is built up from [Mo_2_O_4_F_6_]^4-^ dimers, diprotonated (H_2_dab)^2+^ cations and water molecules (Fig. 1). The Mo_2_O_4_F_6_ unit is formed by two MoO_3_F_3_ octahedra connected by one O–O edge. The MoO_3_F_3_ octahedron is strongly distorted due to the presence of two types of Mo–O bonds: one short bond for the terminal O atom and two medium-ranged bonds for the bridging O atom (Table 1). The Mo–F and Mo–O distances are in good agreement with literature values (Mattes *et al.*, 1980; Adil *et al.*, 2007). Isolated water molecules are hydrogen bonded in a tetrahedral geometry with two –NH_3_ cations and two fluorine atoms (Fig. 2). The inorganic anions and organic cations are connected by intermolecular hydrogen bonds (Fig. 3 and Table 2), creating a two-dimensional network of hydrogen bonds parallel to (-102) between the inorganic anion sheets and organic cation layers.

### Experimental

The starting chemical reactants were molybdenum trioxide (MoO_3_), 1,4-diaminobutane, aqueous HF (48%), water and ethylenglycol in the molar ratio 1:20:55:278:103. The starting mixture was dissolved under solvothermal conditions at 170°C for 72 h. Single crystals were obtained after the evaporation of the solution at room temperature. Crystals suitable for X-ray diffraction were selected under a polarizing optical microscope.

### Refinement

Non-hydrogen atoms were refined with anisotropic thermal factors. H atoms attached to nitrogen atoms were freely refined but their isotropic atomic displacement parameter was constrained to *U*_iso_(H) = 1.5 *U*_eq_(N). Hydrogen atoms attached to carbon atoms were treated in riding motion, with *U*_iso_(H) = 1.2 *U*_eq_(C). The independent H atom of the water molecule was located in a difference Fourier map and was refined using a *SHELXL DFIX* option. Small, unresolved disorder affects the organic cation and consequently, the deepest and highest residual peaks in the final difference Fourier map are located close to carbon atoms. All attempts to decrease these densities using split positions failed.

### Crystal data

**Table:**

(C_4_H_14_N_2_)_2_[Mo_2_O_4_F_6_]·H_2_O	*F*(000) = 568
*M**_r_* = 568.24	*D*_x_ = 1.925 Mg m^−^^3^
Monoclinic, *P*2/*c*	Mo *K*α radiation, λ = 0.71073 Å
Hall symbol: -P 2yc	Cell parameters from 79 reflections
*a* = 8.010 (2) Å	θ = 5.2–61.1°
*b* = 8.788 (2) Å	µ = 1.36 mm^−^^1^
*c* = 14.294 (4) Å	*T* = 296 K
β = 103.019 (12)°	Platelets, orange
*V* = 980.3 (4) Å^3^	0.15 × 0.13 × 0.03 mm
*Z* = 2	

### Data collection

**Table:**

Bruker APEXII diffractometer	3240 independent reflections
Radiation source: fine-focus sealed tube	2690 reflections with *I* > 2σ(*I*)
Graphite monochromator	*R*_int_ = 0.036
ω scans	θ_max_ = 31.6°, θ_min_ = 2.7°
Absorption correction: multi-scan (*SADABS*; Bruker, 2001)	*h* = −11→11
*T*_min_ = 0.816, *T*_max_ = 0.960	*k* = −12→11
35088 measured reflections	*l* = −20→20

### Refinement

**Table:**

Refinement on *F*^2^	Primary atom site location: structure-invariant direct methods
Least-squares matrix: full	Secondary atom site location: difference Fourier map
*R*[*F*^2^ > 2σ(*F*^2^)] = 0.030	Hydrogen site location: inferred from neighbouring sites
*wR*(*F*^2^) = 0.078	H atoms treated by a mixture of independent and constrained refinement
*S* = 1.05	*w* = 1/[σ^2^(*F*_o_^2^) + (0.0354*P*)^2^ + 1.2558*P*] where *P* = (*F*_o_^2^ + 2*F*_c_^2^)/3
3240 reflections	(Δ/σ)_max_ = 0.001
135 parameters	Δρ_max_ = 1.40 e Å^−^^3^
1 restraint	Δρ_min_ = −0.65 e Å^−^^3^
6 constraints	

### Special details

**Table:**

Geometry. All e.s.d.'s (except the e.s.d. in the dihedral angle between two l.s. planes) are estimated using the full covariance matrix. The cell e.s.d.'s are taken into account individually in the estimation of e.s.d.'s in distances, angles and torsion angles; correlations between e.s.d.'s in cell parameters are only used when they are defined by crystal symmetry. An approximate (isotropic) treatment of cell e.s.d.'s is used for estimating e.s.d.'s involving l.s. planes.
Refinement. Refinement of *F*^2^ against ALL reflections. The weighted *R*-factor *wR* and goodness of fit *S* are based on *F*^2^, conventional *R*-factors *R* are based on *F*, with *F* set to zero for negative *F*^2^. The threshold expression of *F*^2^ > σ(*F*^2^) is used only for calculating *R*-factors(gt) *etc*. and is not relevant to the choice of reflections for refinement. *R*-factors based on *F*^2^ are statistically about twice as large as those based on *F*, and *R*- factors based on ALL data will be even larger.

### Fractional atomic coordinates and isotropic or equivalent isotropic displacement parameters (Å^2^)

**Table:**

	*x*	*y*	*z*	*U*_iso_*/*U*_eq_	
Mo1	0.38192 (2)	0.66084 (2)	0.170323 (13)	0.02167 (7)	
F1	0.1200 (2)	0.6202 (2)	0.12852 (14)	0.0432 (4)	
F2	0.3822 (3)	0.6257 (2)	0.02778 (11)	0.0444 (4)	
F3	0.36226 (18)	0.41536 (17)	0.15869 (10)	0.0271 (3)	
O1	0.3694 (2)	0.6283 (2)	0.30531 (12)	0.0262 (3)	
O2	0.3558 (3)	0.8525 (2)	0.15440 (16)	0.0408 (5)	
N1	0.6794 (3)	0.2936 (3)	0.15759 (17)	0.0307 (4)	
H1D	0.781 (5)	0.345 (4)	0.192 (3)	0.046*	
H1E	0.586 (5)	0.322 (4)	0.173 (3)	0.046*	
H1F	0.657 (5)	0.319 (4)	0.097 (3)	0.046*	
C1	0.7020 (4)	0.1273 (3)	0.1650 (2)	0.0407 (7)	
H1A	0.5974	0.0763	0.1327	0.049*	
H1B	0.7292	0.0964	0.2318	0.049*	
C2	0.8514 (4)	0.0847 (4)	0.1166 (3)	0.0453 (7)	
H2A	0.8263	0.1237	0.0515	0.054*	
H2B	0.9559	0.1329	0.1515	0.054*	
C3	0.8797 (4)	−0.0878 (4)	0.1143 (3)	0.0460 (7)	
H3A	0.8566	−0.1331	0.1720	0.055*	
H3B	0.9985	−0.1078	0.1138	0.055*	
C4	0.7625 (4)	−0.1627 (3)	0.0250 (2)	0.0404 (6)	
H4A	0.6441	−0.1347	0.0213	0.049*	
H4B	0.7943	−0.1273	−0.0329	0.049*	
N2	0.7818 (3)	−0.3310 (2)	0.03285 (17)	0.0281 (4)	
H2D	0.894 (5)	−0.360 (4)	0.059 (3)	0.042*	
H2E	0.727 (4)	−0.377 (4)	−0.023 (3)	0.042*	
H2F	0.738 (5)	−0.369 (4)	0.081 (3)	0.042*	
O1W	0.0000	0.4347 (4)	0.2500	0.0432 (7)	
H1W	0.042 (5)	0.496 (4)	0.211 (2)	0.065*	

### Atomic displacement parameters (Å^2^)

**Table:**

	*U*^11^	*U*^22^	*U*^33^	*U*^12^	*U*^13^	*U*^23^
Mo1	0.02193 (11)	0.02317 (10)	0.01799 (10)	0.00157 (7)	0.00045 (7)	0.00148 (7)
F1	0.0233 (8)	0.0492 (10)	0.0505 (11)	0.0027 (7)	−0.0060 (7)	0.0055 (8)
F2	0.0602 (11)	0.0522 (10)	0.0182 (7)	−0.0035 (9)	0.0038 (7)	0.0042 (7)
F3	0.0300 (7)	0.0266 (7)	0.0230 (7)	−0.0014 (5)	0.0021 (6)	−0.0032 (5)
O1	0.0220 (8)	0.0346 (9)	0.0225 (8)	0.0015 (7)	0.0060 (6)	−0.0032 (7)
O2	0.0453 (12)	0.0282 (9)	0.0429 (12)	0.0050 (8)	−0.0030 (9)	0.0073 (8)
N1	0.0350 (12)	0.0347 (11)	0.0232 (10)	0.0056 (9)	0.0084 (9)	0.0011 (9)
C1	0.0411 (16)	0.0312 (13)	0.0463 (17)	0.0048 (11)	0.0023 (13)	−0.0027 (11)
C2	0.0421 (16)	0.0372 (15)	0.056 (2)	0.0025 (13)	0.0096 (14)	−0.0067 (13)
C3	0.0419 (16)	0.0393 (16)	0.0517 (19)	0.0032 (13)	−0.0001 (14)	−0.0096 (13)
C4	0.0449 (16)	0.0310 (13)	0.0400 (16)	0.0029 (11)	−0.0020 (13)	−0.0029 (11)
N2	0.0239 (10)	0.0318 (11)	0.0273 (11)	−0.0008 (8)	0.0029 (8)	−0.0024 (8)
O1W	0.0311 (15)	0.0450 (17)	0.0513 (19)	0.000	0.0043 (13)	0.000

### Geometric parameters (Å, º)

**Table:**

Mo1—O1	1.9754 (18)	C1—H1B	0.9700
Mo1—O1^i^	1.9642 (18)	C2—C3	1.534 (4)
Mo1—O2	1.7058 (19)	C2—H2A	0.9700
Mo1—F1	2.0786 (17)	C2—H2B	0.9700
Mo1—F2	2.0612 (17)	C3—C4	1.551 (4)
Mo1—F3	2.1666 (15)	C3—H3A	0.9700
Mo1—Mo1^i^	2.6126 (7)	C3—H3B	0.9700
O1—Mo1^i^	1.9642 (18)	C4—N2	1.488 (3)
N1—C1	1.473 (4)	C4—H4A	0.9700
N1—H1D	0.96 (4)	C4—H4B	0.9700
N1—H1E	0.87 (4)	N2—H2D	0.93 (4)
N1—H1F	0.88 (4)	N2—H2E	0.91 (4)
C1—C2	1.557 (5)	N2—H2F	0.91 (4)
C1—H1A	0.9700	O1W—H1W	0.895 (10)
			
O2—Mo1—O1^i^	104.84 (9)	N1—C1—H1A	110.2
O2—Mo1—O1	103.97 (9)	C2—C1—H1A	110.2
O1^i^—Mo1—O1	94.50 (7)	N1—C1—H1B	110.2
O2—Mo1—F2	92.49 (9)	C2—C1—H1B	110.2
O1^i^—Mo1—F2	85.79 (8)	H1A—C1—H1B	108.5
O1—Mo1—F2	162.85 (8)	C3—C2—C1	112.3 (3)
O2—Mo1—F1	92.62 (9)	C3—C2—H2A	109.1
O1^i^—Mo1—F1	160.64 (8)	C1—C2—H2A	109.1
O1—Mo1—F1	89.20 (7)	C3—C2—H2B	109.1
F2—Mo1—F1	85.17 (8)	C1—C2—H2B	109.1
O2—Mo1—F3	165.47 (8)	H2A—C2—H2B	107.9
O1^i^—Mo1—F3	85.54 (6)	C2—C3—C4	111.8 (3)
O1—Mo1—F3	84.97 (6)	C2—C3—H3A	109.2
F2—Mo1—F3	77.95 (6)	C4—C3—H3A	109.2
F1—Mo1—F3	75.85 (6)	C2—C3—H3B	109.2
O2—Mo1—Mo1^i^	99.17 (7)	C4—C3—H3B	109.2
O1^i^—Mo1—Mo1^i^	48.64 (5)	H3A—C3—H3B	107.9
O1—Mo1—Mo1^i^	48.28 (5)	N2—C4—C3	109.0 (2)
F2—Mo1—Mo1^i^	134.43 (6)	N2—C4—H4A	109.9
F1—Mo1—Mo1^i^	137.42 (6)	C3—C4—H4A	109.9
F3—Mo1—Mo1^i^	95.32 (4)	N2—C4—H4B	109.9
Mo1^i^—O1—Mo1	83.08 (7)	C3—C4—H4B	109.9
C1—N1—H1D	111 (2)	H4A—C4—H4B	108.3
C1—N1—H1E	112 (2)	C4—N2—H2D	112 (2)
H1D—N1—H1E	114 (3)	C4—N2—H2E	111 (2)
C1—N1—H1F	109 (2)	H2D—N2—H2E	117 (3)
H1D—N1—H1F	111 (3)	C4—N2—H2F	112 (2)
H1E—N1—H1F	100 (3)	H2D—N2—H2F	96 (3)
N1—C1—C2	107.5 (3)	H2E—N2—H2F	108 (3)

Symmetry code: (i) −*x*+1, *y*, −*z*+1/2.

### Hydrogen-bond geometry (Å, º)

**Table:**

*D*—H···*A*	*D*—H	H···*A*	*D*···*A*	*D*—H···*A*
N1—H1*D*···O1*W*^ii^	0.96 (4)	1.93 (4)	2.889 (3)	172 (3)
N1—H1*E*···F3	0.87 (4)	1.94 (4)	2.760 (3)	158 (4)
N1—H1*F*···F2^iii^	0.88 (4)	1.80 (4)	2.679 (3)	178 (4)
N2—H2*D*···F1^iv^	0.93 (4)	1.87 (4)	2.779 (3)	167 (3)
N2—H2*E*···F3^v^	0.91 (4)	1.94 (4)	2.820 (3)	161 (3)
N2—H2*F*···O1^vi^	0.91 (4)	2.00 (4)	2.865 (3)	159 (3)
O1*W*—H1*W*···F1	0.90 (1)	1.82 (1)	2.711 (3)	178 (4)

Symmetry codes: (ii) *x*+1, *y*, *z*; (iii) −*x*+1, −*y*+1, −*z*; (iv) *x*+1, *y*−1, *z*; (v) −*x*+1, −*y*, −*z*; (vi) −*x*+1, *y*−1, −*z*+1/2.
